# Sun Safety: Knowledge and Behavior among Egyptian Farmers—a Multicomponent Intervention Study

**DOI:** 10.1007/s13187-022-02230-3

**Published:** 2022-10-25

**Authors:** Dalia A. El-Shafei, Randa M. Said

**Affiliations:** 1 Department of Community, Environmental and Occupational Medicine, Faculty of Medicine, Zagazig University, Zagazig, Egypt; 2 Department of Family Medicine, Faculty of Medicine, Zagazig University, Zagazig, Egypt

**Keywords:** Sun safety, Knowledge, Behavior, Barriers, Farmer, Vision screening, Brief skin cancer risk assessment tool, Skin cancer screening

## Abstract

The purpose of study was to screen for health hazards related to sun exposure and to examine the effectiveness of a sun safety multicomponent intervention designed specifically for the Egyptian farmers. A multicomponent interventional study was conducted among 128 farmers from three villages in Zagazig district, Sharkia Governorate, Egypt, from January to July 2022. It passed through three phases: phase 1: assessment of participants’ risk of skin cancer and vision screening; phase 2: filling a semi-structured questionnaire assessing sun exposure hazards and sun safety knowledge, behavior, and barriers; and phase 3: conducting multicomponent intervention composed of education session, providing sun safety supplies and reminders then evaluate the effect of this intervention after one month. Most of participants had moderate risk for skin cancer (69.0%), history of photokeratitis (77.3%), cataract (15.6%), and bad/very bad self-reported overall eyesight (43.0%). After intervention, there was a statistically significant improvement in the participants’ awareness regarding sun exposure-related hazards, all knowledge items about sun safety measures (*p* < 0.01) and some sun safety behaviors (*p* < 0.05) including wearing protective clothing, minimization of direct sunlight exposure, taking breaks, plentiful water intake, regular self-checking of skin, wearing wide brimmed hats, and job rotation. Furthermore, there was a statistically significant improvement in all sun protection barriers (*p* < 0.01) except sunscreen and sunglasses related barriers (*p* ˃ 0.05). The multicomponent intervention composed of education session, providing sun safety supplies and reminders was effective in increasing awareness of farmers with sun exposure hazards and improving their knowledge and behavior towards sun safety measures.

## Introduction

Solar ultraviolet radiation (UVR) has been described as a precancerous factor that has a vital role in the causation of skin cancer. Excessive UV exposure can lead also to other negative skin consequences, such as actinic keratosis and premature skin aging [1]. There is also a cause-effect relationship between UVR exposure, and many eye problems such as photokeratitis, pinguecula, pterygium, cataract, and possibly ocular neoplasm [2]. Furthermore, exposure to UVR leads to worldwide loss of 1.5 million days from people’s lives each year when disability-adjusted life year rates (DALY) was calculated [3]. Despite all previously mentioned health hazards, the hazard of regular exposure to UVR has completely undiscovered until now [4].

Outdoor workers are exposed to solar UVR nearly eight times more than indoor workers. So, they are at increased risk for the development of solar UVR-related health hazards. Occupational exposure to UVR among several outdoor occupations (e.g., construction and agricultural sector) was estimated in numerous UV dosimetry studies to be greater than the safe limits determined by the International Commission on Non-Ionizing Radiation Protection (ICNIRP) [2]. So, our study would pay attention towards farmers because they spend most of their working hours outdoors, putting them at increased risk of health problems related to sunlight exposure. Furthermore, although they are not exposed only to sunlight risks but also to a diversity of environmental risk factors such as chemicals, mechanical devices, and various biological risks, their occupation has less safety protocols than other occupations [5].

Our study would also pay attention to promote sun safety behaviors because occupational exposure to solar UV radiation is a modifiable risk factor with high possibility for change in workplaces. This is consistent with the recent reviews and recent Community Preventive Services Task Force recommendation which reported strong evidence for the effectiveness of targeted interventions in promoting outdoor workers’ sun safety habits. These interventions were multicomponent; i.e., they were not only limited to education sessions aimed to increase the workers’ awareness of sun exposure-related health hazards and how to avoid them but also included various strategies such as providing wide brimmed hats and sunscreen, distributing reminders of sun safety, training of safety officers, developing policy and safety regulations, and applying role modeling through peer leader [1].

Sun safety programs directed to manual outdoor workers especially in agriculture and construction sectors have achieved successful improvements in knowledge, attitudes, and practice. Nevertheless, according to our knowledge, there is a lack in sun safety intervention research studies targeting the Egyptian agriculture workers. So, increasing the research on sun safety, especially in sunny countries like Egypt, is essentially an urgent need. Therefore, the aim of this study was to screen for health hazards related to solar UVR exposure and to examine the effectiveness of a sun safety multicomponent intervention designed specifically for the Egyptian farmers.

## Materials and Methods

A multicomponent interventional study was conducted among farmers living in three villages of Zagazig district, Sharkia Governorate, Egypt, from January to July 2022.

### Study Population

#### Determination of Sample Size and Sampling Technique

The sample size was estimated to be 116 subjects using the paired *t* statistic online calculator depending on effect size 50%, standard deviation of the change in the outcome 1.9 according to result of previous study [6], a confidence interval of 95%, and the power of test 80%, taking into consideration 20% dropouts, so the calculated sample size was 139 subjects, of which 128 of them completed the educational intervention.

A two-stage cluster sample was used. In the 1st stage, three villages were selected by simple random sampling technique from all villages of Zagazig district. The 2nd stage included the selection of farmers. The researchers started at the center of the selected village and randomly selected a direction. They started at the nearest farmland then through farmland-to-farmland survey; they interviewed the farmers consecutively until they reached the target sample size. The sample size was divided equally over the three villages (46 farmers in each).

Inclusion criteria included all farmers over 18 years of age who consented to participate in the study. Exclusion criteria included farmers with previous diagnosis of skin cancer, received phototherapy for treatment of dermatological condition, suffering from an intellectual disability or mental impairment of any kind.

The pilot study was conducted on 10% of the sample (13 farmers) during January 2022 to test the applicability of the study and to test the response to different items of the questionnaires. The reliability for all questionnaires was acceptable with Cronbach’s alpha coefficients ranged from 0.72 to 0.94. According to the result of the pilot study, some modifications were done on questionnaires. All farmers of the pilot were excluded from the results of the study.

### Study Procedures and Data Collection Tools

Our study was conducted in three phases:

- **Phase 1**: Included assessment of participants’ risk of skin cancer and vision screening through the following:
  - **Brief skin cancer risk assessment tool (BRAT)**: It is a valid and reliable tool adapted from Glanz et al. (2015) [7] addressing the key risk factors for skin cancers. It was an interviewer administered. Its score was based on asking about mole count (none, 0; 1–2, 5; 3–5, 10; 6–10, 20; > 10, 30), freckles count (none, 0; few, 2; many, 4), childhood residence (all participants were from tropics, 10), sunburn history (none, 0; 1–2, 1; 3–5, 2; 6–10, 3; > 10, 4), skin color (dark brown/black, 0; med brown, 2; light brown, 4; olive,16; fair, 18; very fair, 20), natural hair color (black, 0; dark brown, 1; light brown, 2; blonde, 3; red, 4), ease of sun burning (no, 0; yes, 3), and tanning (dark, 0; medium, 1; light, 2; none, 3). Scores on the BRAT ranged from 0 to 78. Participants scoring 26 or below were placed in the low-risk category; scores of 27–35 were considered moderate-risk; and scores of 36 and higher were classified as high-risk.
  - **Vision screening**: Screening for eye problems linked to sun exposure was done through the following approach: firstly, asking about symptoms of photokeratitis (pain or redness in the eyes, swelling, light sensitivity, blurry vision, gritty sensation in the eyes, twitching of the eyelids, temporary loss of vision and seeing halos that may last from 6 to 24 h, but they usually disappear within 48 h). Secondly, spot diagnosis for pinguecula, pterygium, and cataract. Finally, screening for visual impairment due to sun exposure induced cataract or macular degeneration by a self-reported visual function questionnaire adapted from Quandt et al. (2016) [5]. It was an interviewer-administered asking the farmers to rate their eyesight using both eyes as very good, good, moderate, bad, or very bad and how much difficulty they had in four activities needing far or near vision: (1) recognizing a friend across the street, (2) watching television, (3) reading print, and (4) doing work or hobbies that require near vision. The five responses to each activity were none, mild, moderate, severe, and extreme/cannot do.
- **Phase 2**: After giving the participants feedback about their risk of skin cancer and the results of vision screening, they were informed about the need for an educational intervention for sun safety. In this phase, a semi-structured pre-test questionnaire guided by relevant literature and previous studies [6, 8] was distributed among farmers. After explaining the purpose of the study, the pre-test questionnaire was filled through a face-to-face interview with farmers and took 10–15 min to be completed. All participants were asked questions covering the following sections:
  - **Section I**: Sociodemographic and occupational characteristics of the studied farmers include age, gender, marital status, level of education, family history of skin cancer, duration of work, the average number of hours spent working outside per day, and training received on the risks of sun exposure in last 12 months.
  - **Section II**: Assessing sun exposure hazards and sun safety knowledge of studied farmers by 21 items divided into six items assessing awareness of sun exposure-induced skin damage (skin burn, tanning, skin aging, hyperpigmentation, skin cancer, no side effects), other six items assessing awareness of sun exposure-induced eye damage (aging, corneal sunburn, cataracts, pinguecula and pterygia, macular degeneration, no side effects), and nine items assessing knowledge about sun safety measures “sunscreen (2 items), sunglasses (1 item), time of sun exposure (2 items), wearing a hat (1 item), clothing (1 item), tanning (2 items).” Participants indicated their agreement with each statement on a 5-point Likert scale ranging from 1 = strongly disagree to 5 = strongly agree.
  - **Section III**: Assessing sun safety practice of the studied farmers through measuring 11 behaviors (using shade, protective clothes, sunglasses, sunscreen, plentiful water intake, checking the UV index for the day, regular self-checking of skin, limiting time in sun between 10 am and 4 pm, wearing a hat, and job rotation). Frequency of use for each sun protection behavior was rated using a 5-point Likert scale ranging from 1 = rarely to 5 = always.
  - **Section IV**: Assessing barriers to sun protection behaviors represented in seven items include lack of knowledge about sun protection measures, lack of concern about sun exposure, feeling too hot when wearing sun protection clothing, inconvenience of sun self-protection all the time, expensive sun protection measures, sunscreen-related barriers (use of sunscreen is too feminine, I do not like the smell of sunscreen, sunscreen is greasy, sunscreen attracts dirt, and I cannot reapply sunscreen), and sunglasses related barriers (glasses fall off the face while farming, I feel it affect my vision). Participants reported their agreement with each statement on a dichotomous yes/no scale with addition of a third response “not applicable.” The frequency of agreement is compared before and after intervention.
- **Phase 3**: A multicomponent health education program was conducted after assessing the gap in the studied farmers’ knowledge and behavior towards sun safety from analysis of the pre-test questionnaire. The educational message was tailored to satisfy the needs of the studied farmers and to remove barriers to sun protection behaviors. The objectives of the program were to:
  - Increase the farmers’ perceived risks of sun exposure on skin and eye.
  - Promote the farmers sun safety practices including using shade when working in the sun, wearing long-sleeved loose-fitting tops and trousers with an ultraviolet protection factor (UPF) rating of ≥ 30, wearing wraparound-style sunglasses that block out 99% to 100% of UVR, using sunscreen with a sun protection factor [SPF] ≥ 30 in correct way, wearing a wide brimmed hat with neck protection instead of the beloved baseball hats, plentiful water intake, checking the UV index for the day through the weather app on their smart phone, regular self-checking of skin for moles or unusual changes and seeking medical advice immediately, minimization of direct sunlight exposure in middle of day between “10 AM to 4 PM” through doing much of work before 10 AM or after 4 PM, taking breaks, and job rotation.
  - Eliminate barriers to sun protection behaviors through (1) providing samples of sunglasses, sunscreens, and wide brimmed hats; (2) marketing to cheap and at the same time effective types of sunscreen and sunglasses; (3) demonstrating how to fix sunglasses to avoid its fall off the face during farming; (4) Advising to have glasses designed for both sight correction and sun protection at the same time; (5) Providing list of clothes choices suitable for sun protection; and (6) offering various options to minimize direct sunlight exposure in middle of day.

According to Egyptian General Authority of Meteorology (2022) [9], the months with the highest UV index in Egypt are June and July (UV index 12), while the month with the lowest UV index is December (UV index 3). So, the time of the intervention was chosen to be in April and May between 9 AM and 6 PM (working hours). Participants were divided into three groups. Each group included the farmers living in the same village. Each participant was asked to attend one training session of 1-h period. The session was held during the time preferred by the participants. Different training methods were used for illustration as the PowerPoint presentation, photos, and video files. At the end, participants were given flyers containing all needed information to ensure remembering the message. One month after the implementation of the training program, the participants were asked to complete the same questionnaire that was used in the pre-test to detect the effect of the training program.

### Statistical Analysis

The collected data were statistically analyzed using SPSS program (Statistical Package for Social Science) version 16.0. Qualitative data were represented as frequencies and percentages and were compared using McNemar’s test. Quantitative data were represented as mean ± SD and were compared using Paired *t*-test. The test results were considered significant when p value < 0.05. Excel 2019 was used to draw the graphs.

## Results

One hundred and twenty-eight farmers were included in the study. More than half of them were males (56.3%) and less than 40 years old (61.7%) with a mean age of 28.1 ± 8.9 with basic education (51.6%). Most of them were married (68.0%). Only 5 of them (3.9%) had positive history of family member skin cancer. As regards occupational history, more than half of participants (66.4%) had worked in farming for more than 10 years with a mean of 21.9 ± 8.3 with an average number of hours worked outdoor on a typical day of 9.4 ± 3.5. Only 11.7% of them received sun safety training in last 12 months (Table 1).

**Table 1:** Socio-demographic and occupational characteristics of the studied farmers

Characteristics	(*N* = 128) No	%
Age groups		
< 40 years	79	61.7
≥ 40 years	49	38.3
Mean ± SD	28.1 ± 8.9	
Range	19–68	
Gender		
Male	72	56.3
Female	56	43.7
Marital status		
Un-married*	41	32.0
Married	87	68.0
Level of education		
Illiterate	45	35.2
Basic education	66	51.6
University/technical diploma	17	13.3
Family member had skin cancer		
Yes	5	3.9
No	123	96.1
Duration of work (years)		
< 10 yrs	43	33.6
≥ 10 yrs	85	66.4
Mean ± SD	21.9 ± 8.3	
Range	3–42	
Hours worked outdoors on a typical day		
Mean ± SD	9.4 ± 3.5	
Range	6–12	
Sun safety training received in last 12 months		
Yes	15 (11.7)	
No	113 (88.3)	

*Un-married including single, divorced, and widower

**Graph 1:**
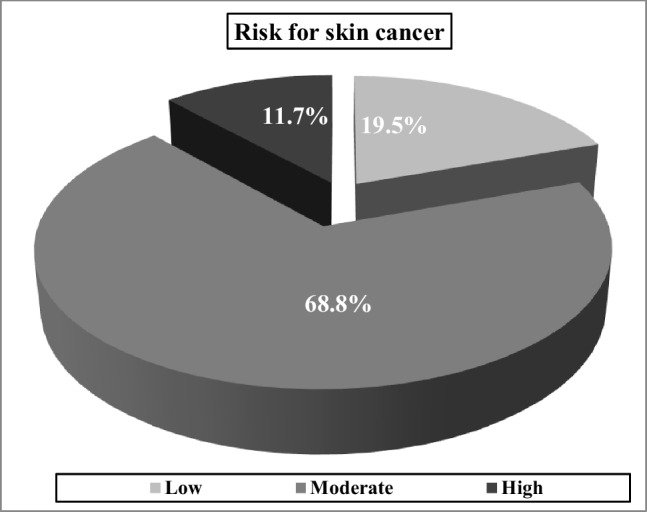
Risk of skin cancer among studied farmers

The assessment of participants’ risk of skin cancer through brief skin cancer risk assessment tool (BRAT) revealed that most of participants (69.0%) had moderate risk for skin cancer (Graph 1).

As regarding vision screening, Table 2 illustrates that most participants (77.3%) had a history of photokeratitis. About one quarter of them (24.2%) had pinguecula on spot diagnosis. The overall self-reported eyesight was bad/very bad among near half of participants (43.0%). Moderate difficulty was the most prevalent in all evaluated activities “recognizing a friend across the street (46.1%), watching television (48.4%), reading fine print (46.1%), doing work or hobbies requiring up close vision (48.4%).”

**Table 2:** Results of vision screening among studied farmers

Items	(*N* = 128) No	%
History of photokeratitis	99	77.3
Spot diagnosis		
Pinguecula	31	24.2
Pterygium	13	10.2
Cataract	20	15.6
Overall self-reported eyesight		
Very good/good	32	25.0
Moderate	41	32.0
Bad/very bad	55	43.0
Difficulty recognizing a friend across the street		
None/mild	39	30.5
Moderate	59	46.1
Severe/extreme or cannot do	30	23.4
Difficult watching television		
None/mild	50	39.1
Moderate	62	48.4
Severe/extreme or cannot do	16	12.5
Difficulty reading fine print		
None/mild	51	39.8
Moderate	59	46.1
Severe/extreme or cannot do	18	14.1
Difficulty doing work or hobbies requiring up close vision		
None/mild	34	26.6
Moderate	62	48.4
Severe/extreme or cannot do	32	25.0

Results of this study showed that there was a significant improvement in the participants’ awareness (pre-/post-intervention) regarding sun exposure-induced skin and eye damages (*p* < 0.01). As regards the knowledge about sun safety measures, it was significantly improved (*p* < 0.01) after intervention in all items (Table 3).

**Table 3:** Awareness of sun exposure hazards and knowledge about sun safety measures among studied farmers

Items	Pre-intervention	Post-intervention	*p* value
Sun exposure-induced skin damage	44.92 ± 7.42	93.63 ± 15.01	0.000*
Sun exposure-induced eye damage	36.88 ± 9.34	84.46 ± 13.39	0.000*
Sun safety measures			
- Sunscreen	48.27 ± 7.38	91.55 ± 15.24	0.000*
- Sunglasses	38.98 ± 8.14	87.42 ± 14.31	0.000*
- Time of sun exposure	49.67 ± 9.32	92.15 ± 13.10	0.000*
- Wearing a hat	43.68 ± 9.02	91.46 ± 14.69	0.000*
- Clothing	40.39 ± 7.19	89.51 ± 13.87	0.000*
- Tanning	42.77 ± 8.75	90.72 ± 11.36	0.000*

^*^Highly statistically significant (*p* < 0.01)

As regards assessing sun safety practice through sun protection behavior index (pre/ post Intervention), it was significantly improved after intervention (*p* < 0.05) especially in the items of clothing type, minimization of direct sunlight, taking breaks, plentiful water intake, regular self-checking of skin, wearing wide brimmed hats with neck protection, and job rotation (Table 4).

**Table 4:** Sun safety practice through sun protection behavior index among studied farmers

Items	Pre-intervention	Post-intervention	*p* value
Using a shade/cover when working in the sun	36.35 ± 11.23	38.71 ± 12.08	0.067
Clothing type	32.27 ± 9.64	51.32 ± 11.37	0.005*
Minimization of direct sunlight	38.25 ± 11.59	57.44 ± 13.03	0.014*
Taking breaks	38.67 ± 11.23	45.07 ± 11.84	0.049*
Wearing sunglasses	22.72 ± 10.84	27.42 ± 13.89	0.071
Using sunscreen in correct way	22.65 ± 9.13	29.19 ± 9.33	0.061
Plentiful water intake	45.75 ± 13.44	61.83 ± 13.58	0.009*
Check the UV index for the day	17.09 ± 8.87	21.21 ± 9.33	0.089
Regular self-checking of skin	35.50 ± 11.82	53.78 ± 11.32	0.011*
Wearing wide brimmed hats with neck protection	59.54 ± 9.42	75.54 ± 10.35	0.012*
Job rotation	41.87 ± 10.09	46.43 ± 8.69	0.023*

^*^Statistically significant (*p* < 0.05)

**Graph 2:**
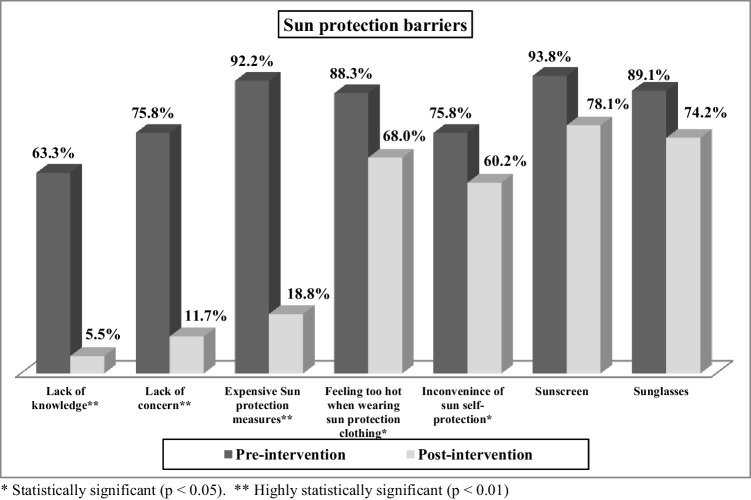
Evaluation of sun protection barriers among the studied farmers

Graph 2 illustrates that there was a statistically significant improvement in the participants’ agreement with the sun protection barriers among the studied farmers (pre-/post-intervention), especially in the items of lack of knowledge or concern, and expensive sun protection measures (*p* < 0.01).

## Discussion

According to the results of screening phase of this study, most of farmers (69.0%) had moderate risk for skin cancer according to BRAT. This is consistent with many studies which concluded that due to the major role of sun exposure in skin cancer especially early and long-term exposure, outdoor workers—primarily farmers—are at significantly higher risk of developing skin cancer [10, 11]. The results of screening also revealed many eye problems among the studied farmers either acute problems like photosensitivity in the most of them or chronic problems like pinguecula, pterygium and cataract in fair number of them. Furthermore, there was moderate difficulty in self-reported overall eyesight, near vision, and far vision which may be due to sun exposure-related cataract or macular degeneration. This is consistent with many studies which described the effects of solar UVR on the eye [12, 13].

Regarding the change in knowledge and behavior of the studied farmers towards sun safety after intervention, although the study showed significant improvement in all knowledge items, it only showed significant improvement in some sun safety behaviors such as using protective clothes, taking breaks, plentiful water intake, regular self-checking of skin, wearing wide brimmed hats with neck protection, and job rotation. This is consistent with the narrative review which concluded that face-to-face educational interventions, such as those demonstrated in Utah, Turkey, and Michigan, proved to be highly effective in changing knowledge, attitude, and behavior of farmers towards sun safety measures [10].

The sun safety behaviors that did not show improvement after intervention included using a shade when working in the sun, wearing sunglasses, using sunscreen, and checking the UV index for the day. This could be explained by the self-reported barriers that the study tried to eliminate but resulted in insignificant improvement. These barriers included: Firstly, the barrier against using a shade when working in the sun was “this behavior is not working for farmers.” Secondly, the barrier against wearing sunglasses was “this behavior is expensive and impractical because of repeated fall of sunglasses from the face during farming and sunglasses cannot replace eyeglasses if vision is impaired.” Thirdly, the barrier against using sunscreen was “this behavior is expensive, the use of sunscreen is too feminine, its smell is not liked, its nature is greasy, it attracts dirt, and the user needs to reapply it to work correctly.” Finally, the barrier against checking the UV index for the day was “most of the studied farmers were illiterate or of basic education so, they could not use the weather app on their smart phone.” Many studies conducted in Australia, Brazil, and the USA reported that the majority of farmers recognized wearing a wide brimmed hat as their primary method of sun protection, while dependence on other sun protection practices, including wearing sunglasses or using sunscreen, was much lower [14–16].

The self-reported barriers to the sun protection behaviors that showed a statistically significant improvement among the studied farmers after intervention included lack of knowledge about sun protection practices, lack of concern about sun exposure, feeling too hot when wearing sun protection clothing, and inconvenience of sun self-protection all the time. The study succeeded to eliminate all these barriers through tailoring specific messages for farmers providing them with practical solutions. These barriers reflect that the Egyptian agriculture sector is neglected and miss the opportunities of the educational interventions that raise the awareness with occupational health hazards. So, attention should be directed towards designing educational interventions specifically for farmers. Further, tailoring the message content of these interventions may have greater impact on farmers than generic or non-tailored messages as such messages are seen as more personally relevant, and consequently may be more probable to motivate its recipients to make healthful behavior changes [7]**.**

Finally, this study showed that although the studied farmers became aware of the risks posed by excessive sun exposure, they typically did not perform routinely all the sun protective behaviors especially that needed financial support such as using sunglasses and sunscreen. This reflects the importance of organizational role in the protection of farmers from the sun through providing the safety supplies [1]. The relation between the perceived risk posed by excessive sun exposure and positive sun protection behaviors among farmers is controversial where some studies concluded that a higher perception of risk has been found to be strongly associated with more positive sun protection behaviors [17], while other studies concluded that although farmers were aware of the dangers posed by excessive sun exposure, they typically did not wear adequate protection when working outdoors [10].

Limitations of the study include the following: first, it was one group pretest–posttest study with no control group, a study design that might identify ineffective program, but could not confirm the cause-effect relationships between program and outcome; second, lack of long-term follow-up that may be needed to assure the maintenance of preventive behaviors; third, inability to generalize the results because of small sample size which necessitates further studies; and finally, in a self-reported study, participants may over-report sun safety practices leading to inflated results. Despite its limitations, this study constitutes an important first step in research focused on the development of knowledge and understanding concerning sun safety issues in the Egyptian agriculture sector.

## Conclusion and Recommendations

Based on the results, the multicomponent intervention composed of education session, providing sun safety supplies and reminders, was effective in increasing awareness of farmers with sun exposure hazards and improving their knowledge and behavior towards sun safety measures. So, it is recommended that more aggressive educational interventions in the form of agricultural events, healthcare partnerships, and promotional sun safety campaigns should target farmers as a reminder to protect themselves against the hazards of sun exposure. These interventions should also assure providing farmers with sun safety supplies to motivate them to adhere to the sun protective behaviors.

## Data Availability

The study tools; including used survey questions and informational materials are available from the corresponding author upon request.
